# Aspects of work and sleep associated with work ability in regular aviation pilots

**DOI:** 10.11606/S1518-8787.2019053000345

**Published:** 2019-01-18

**Authors:** Pollyanna Pellegrino, Elaine Cristina Marqueze

**Affiliations:** I Universidade Católica de Santos. Programa de Pós-Graduação em Saúde Coletiva. Santos, SP, Brasil; II Universidade Católica de Santos. Departamento de Epidemiologia. Programa de Pós-Graduação em Saúde Coletiva. Santos SP, Brasil

**Keywords:** Pilots, Work Ability Evaluation, Sleep, Working Conditions, Occupational Health, Pilotos, Avaliação da Capacidade de Trabalho, Sono, Condições de Trabalho, Saúde do Trabalhador

## Abstract

**OBJECTIVE:**

Analyze the association of work organization and sleep aspects with work ability in regular aviation pilots.

**METHODS:**

This is a cross-sectional epidemiological study with 1,234 regular aviation pilots who worked domestic and international flights, affiliated with the Brazilian Association of Civil Aviation Pilots. Data collection employed online questionnaire. We compared proportions using Pearson's Chi-squared or Fisher's exact hypothesis tests. Then, we conducted Poisson analysis, with robust variance, to test factors associated with moderate or low work ability.

**RESULTS:**

The prevalence of moderate or low work ability was 43.3%. We found that self-perception of insufficient sleep (PR = 1.29; 95%CI 1.06–1.57), increased perception for fatigue (PR = 1.51; 95%CI 1.24–1.84), more than 65 flight hours per month (PR = 1.22; 95%CI 1.01–1.46), less than 10 days of time off per month (PR = 1.27; 95%CI 1.04–1.55), and frequent operational delays (PR = 1.23; 95%CI 1.02–1.48) were factors associated with moderate or low work ability.

**CONCLUSIONS:**

Work organization was a determining factor for decreased work ability, especially concerning aspects related to rest and its influence on the sleep of pilots.

## RESUMO

**OBJETIVO:**

Analisar a associação da organização do trabalho e dos aspectos do sono com a capacidade para o trabalho entre pilotos da aviação regular.

**MÉTODOS:**

Foi realizada uma pesquisa epidemiológica transversal, com 1.234 pilotos da aviação regular, que realizavam voos de rotas nacionais e internacionais, afiliados à Associação Brasileira de Pilotos da Aviação Civil. A coleta de dados foi feita através de questionário *on-line*. Para comparação entre as proporções foram realizados os testes de hipóteses qui-quadrado de Pearson ou Exato de Fisher. Posteriormente, foi efetuada a análise de Poisson, com variância robusta, para testar os fatores associados à capacidade moderada ou baixa para o trabalho.

**RESULTADOS:**

A prevalência de capacidade para o trabalho moderada ou baixa foi de 43,3%. Verificou-se que autopercepção de sono insuficiente (RP = 1,29; IC95% 1,06–1,57), maior percepção para fadiga (RP = 1,51; IC95% 1,24–1,84), jornada de voo maior que 65 horas por mês (RP = 1,22; IC95% 1,01–1,46), menos de 10 dias de folga por mês (RP = 1,27; IC95% 1,04–1,55) e atrasos operacionais frequentes (RP = 1,23; IC95% 1,02–1,48) foram fatores associados à capacidade moderada ou baixa para o trabalho.

**CONCLUSÕES:**

A organização do trabalho foi um fator determinante para a diminuição da capacidade para o trabalho, em especial quanto aos aspectos relacionados ao descanso e suas repercussões no sono de pilotos.

## INTRODUCTION

In recent decades, regular aviation has grown significantly and is part of a process of transformation of the production system, with changes also in the organization and management of air transport^9^. These data demonstrate the importance of the sector for the economy, in air transportation of both passengers and freight^9^. According to the Air Transport Yearbook of the Brazilian National Civil Aviation Agency (ANAC), the number of paid passengers transported by plane in Brazil more than doubled in 10 years, from 26.8/100,000 inhabitants in 2005 to 58.7/100,000 inhabitants in 2014. On the other hand, the number of pilots of Brazilian companies grew only 21% over this period^a^. Such increased demand with no increase in staff has been negatively associated with the health of pilots, which is regulated by civil aviation standards and is responsibility of the State^9^.

This new context in aviation leads pilots to overloading, higher frequency of changes in schedule, psychological pressure, and stress, affecting their health and endangering the safety of flights^14^. However, these professionals need training and capacity to perform their job. Ilmarinen^8^ argues that this ability is a process of interaction between the resources of human beings and their work. Worker-related resources are their functional capacities; physical, mental, and social health; education; competence; values; attitudes and motivation. This is a dynamic process, which undergoes numerous changes over work life, mainly related to functional aging^8^.

Studies with airline pilots show that this category typically presents several sleep and health problems, especially excessive sleepiness, unintentional naps, and fatigue^14^ ^,^ ^20^ ^,^ ^23^. However, such studies are still incipient, and knowing characteristics of the work and health of these professionals is necessary to enable the action of public policies and make prevention and health promotion activities effective.

In this context, this article aims to analyze the association of work organization and sleep aspects with work ability in regular aviation pilots.

## METHODS

Cross-sectional epidemiological study, with pilots (captains and copilots) of Brazilian regular aviation, of domestic and international flights routes, affiliated with the Brazilian Association of Civil Aviation Pilots (ABRAPAC). All pilots affiliated with the ABRAPAC (2,530) were invited to participate in the study using email and 1,234 participated in the study (48.8%). According to the ANAC^a^, the number of pilots associated with the ABRAPAC represented approximately half of the Brazilian regular aviation pilots at the time of this study (5,956 pilots). It should be noted we used convenience sampling, since only pilots associated with the ABRAPAC were invited to participate in the study.

Initially, sample size was estimated to meet the objectives of the research “*Fadiga crônica, condições de trabalho e saúde de pilotos brasileiros*” [Chronic fatigue, working conditions, and health of Brazilian pilots]^b^, with which this study is associated. Thus, sample power was estimated *a posteriori* (G*Power 3.1.4 software), to evaluate the internal validity of the results found in this study. We found 99% sample power, with 95% confidence interval (α = 5%) to detect prevalence ratios equal to or greater than 1.20 as significant, having as parameter the prevalence of moderate or poor work ability (43.3%).

Study variables were: sociodemographic characteristics and work, sleep, and lifestyle aspects.

* Sociodemographic characteristics:
  - Sex;
  - Age, dichotomized from mean age;
  - Educational level.
* Work aspects:
  - Current function;
  - Flight routes;
  - Monthly flight hours, dichotomized from mean;
  - Average days off per month;
  - Maximum number of consecutive days of work in the last six months, dichotomized from mean;
  - Maximum number of consecutive nights of work in the last six months;
  - Frequency of operational flight delays;
  - Time working as pilot, dichotomized from mean;
  - Time working in the night shift;
  - Quality of place for rest on plane, dichotomized from mean;
  - End time of night shift;
  - Need for recovery after work (evaluated using the scale proposed by Veldhoven and Broersen26, with the 0–100 points score classified by tercile: less, moderate, and greater need for recovery) (Cronbach's alpha 0.81);
  - Occupational stress was evaluated by the demand, control, and social support questionnaire Job Stress Scale, using the version adapted to Portuguese2. This questionnaire evaluates the occupational stressors and according to the demands and the control is categorized into: low job strain (high control and low demand), high job strain (low control and high demand), passive work (low control and low demand), and active work (high control and high demand). Cronbach's alpha for the demand questionnaire was 0.72, for control 0.50, and for social support 0.81.
* Sleep and lifestyle aspects:
  - Perception of chronotype, evaluated using a single question taken from the Karolinska sleep questionnaire^1^;
  - Perception of sufficient sleep, evaluated using a single question taken from the Karolinska sleep questionnaire^1^;
  - Perception of sleep quality, evaluated using a single question taken from the Karolinska sleep questionnaire^1^;
  - Chance of developing obstructive sleep apnea syndrome, evaluated using the Berlin questionnaire^17^ (Cronbach's alpha 0.67);
  - Excessive sleepiness, evaluated using the Epworth sleepiness scale^3^ (Cronbach's alpha 0.81);
  - Perception of fatigue, evaluated using the Yoshitake questionnaire^27^ (Cronbach's alpha 0.93);
  - Unintentional nap during work, evaluated using a single question taken from the Karolinska sleep questionnaire^1^;
  - Symptoms of insomnia, evaluated using seven questions of the Karolinska Sleep Questionnaire^1^, index proposed by Nordin et al.^18^ (Cronbach's alpha 0.88);
  - Weekly physical activity time, categorized into ≥ 150 minutes/week or < 150 minutes/week^19^.

To evaluate work ability (dependent variable), we used the version translated to Portuguese^25^ of the work ability index (WAI), which is based on the individuals’ self-perception of their work ability (Cronbach's alpha 0.82). By estimating the score, the work ability of all pilots was classified into great (45–49 points), good (41–44 points), moderate (37–40 points), or low (7–36)^10^. These cut-off points were adopted because the sample under study is essentially composed of young adult workers (74.2% of the pilots were aged up to 45 years) and this research aims to discuss the factors associated with low or moderate work ability, so preventive actions can be proposed based on the results. It should be noted that, for the Poisson regression analysis, we grouped the categories low or moderate (tested) and good or great (reference).

The variables were described using absolute and relative frequencies. We compared proportions using Pearson's Chi-squared or Fisher's exact hypothesis tests. Given the type of study (cross-sectional) and the high prevalence of the outcome, we chose Poisson regression, with robust variance, to analyze the factors associated with moderate or low work ability. Independent variables with p < 0.20 in the hypotheses tests were tested in the multiple model, in decreasing order of statistical significance (stepwise backward technique). The model was adjusted for age and sex variables, because these variables showed no differences between the proportions of work ability. The significance level adopted in all tests was 5%. We used Stata 12.0 (Stata corp, Texas, USA) for statistical analyses.

This study was approved by the Research Ethics Committee of the Federal Institute of Education, Science, and Technology of São Paulo (Protocol 625,158).

## RESULTS

Most pilots interviewed were male (97.1%), aged under 39 years (52.4% – mean age 39.1 years, SD = 9.8 years), with educational level above that required for exercise of the profession (71.3%), had a partner (84.4%), did not live in the same location of their contractual basis (53.8%), and held the position of domestic pilot (51.7%), followed by domestic copilots (39.1%). The prevalence of moderate or low work ability was 43.3%.

Compared with pilots with great or good work ability, there was a higher proportion of pilots with moderate or low work ability who practiced less than 150 minutes of physical activity per week, who reported sleeping insufficiently and sleeping very poorly, who presented high chance of developing obstructive sleep apnea, with excessive sleepiness, with higher perception of fatigue, who presented unintentional naps during work, and who presented symptoms of insomnia. While as to occupational variables, the group with moderate or low work ability showed higher proportion of pilots with function of domestic and international captain, who worked in international flight routes, with more monthly flight hours than the group average, with less than nine days off per month, with seven consecutive days of work or more, who often had operational delays, who had worked longer in the profession and more time on the night shift, who assessed the quality of the place of rest on the plane as poor, who ended the night shift too late, with greater need for recovery after work and with a work classified as active and also low job strain. All these differences were statistically significant (p < 0.05) (Table 1).

**Table 1:** Work ability related to sociodemographic, lifestyle, sleep, and work characteristics. Brazil, 2017.

Variable	Category	Great or good work ability	Moderate or low work ability	P
		n (%)	n (%)	
Sex	Female	22 (3.1)	14 (2.6)	0.59
	Male	678 (96.9)	520 (97.4)	
Age	≤ 38 years	380 (54.5)	264 (49.6)	0.08
	≥ 39 years	317 (45.5)	268 (504)	
Educational level	complete or incomplete graduate program	84 (12.0)	53 (9.9)	0.48
	complete or incomplete undergraduate program	492 (70.3)	388 (72.7)	
	Complete high school	124 (17.7)	93 (17.4)	
Weekly physical activity time	150 minutes or more	382 (54.6)	245 (46.0)	< 0.01
	Less than 150 minutes	317 (45.4)	288 (54.0)	
Perception of chronotype	Indifferent	92 (13.2)	49 (9.2)	0.08
	Morningness or extreme morningness	276 (39.4)	215 (40.2)	
	Eveningness or extreme eveningness	332 (47.4)	270 (50.6)	
Perception of sufficient sleep time	Yes	541 (77.3)	309 (57.9)	< 0.01
	No	159 (22.7)	225 (42.1)	
How well do you think you sleep	Very well or well	434 (62.0)	206 (38.6)	< 0.01
	Neither well, nor poorly	228 (32.6)	235 (44.0)	
	Fairly or very poorly	38 (5.4)	93 (17.4)	
Obstructive sleep apnea syndrome	Low chance	601 (85.9)	382 (71.5)	< 0.01
	High chance	99 (14.1)	152 (28.5)	
Sleepiness (Epworth)	Low sleepiness	453 (64.7)	264 (49.4)	< 0.01
	Excessive sleepiness	247 (35.3)	270 (50.6)	
Perception of fatigue	Lower fatigue	557 (84.6)	304 (60.7)	< 0.01
	Higher fatigue	101 (15.4)	197 (39.3)	
Unintentional nap during work	No	364 (52.0)	157 (29.4)	< 0.01
	Yes	336 (48.0)	377 (70.6)	
Symptoms of insomnia	Without symptoms	391 (56.9)	170 (32.5)	< 0.01
	With symptoms	296 (43.1)	353 (67.5)	
Current function	International captain	29 (4.1)	48 (9.0)	< 0.01
	Domestic captain	347 (49.6)	291 (54.5)	
	International copilot	17 (2.4)	19 (3.6)	
	Domestic copilot	307 (43.8)	176 (32.9)	
Flight routes	Domestic	654 (93.4)	467 (87.5)	< 0.01
	International	46 (6.6)	67 (12.5)	
Monthly flight hours	Up to 65 hours	356 (51.5)	217 (41.1)	< 0.01
	66 hours or more	335 (48.5)	311 (58.9)	
Average days off per month	10 days or more	338 (48.3)	176 (33.0)	< 0.01
	Up to 9 days	362 (51.7)	357 (67.0)	
Maximum number of consecutive days of work	Up to 6 days	591 (84.8)	408 (77.3)	< 0.01
	7 days or more	106 (15.2)	120 (22.7)	
Maximum number of consecutive nights of work	One or two nights	122 (18.1)	82 (15.9)	0.19
	Three or four nights	386 (57.4)	283 (55.1)	
	Five nights or more	165 (24.5)	149 (29.0)	
Frequency of of operational flight delays	Never, rarely or sometimes	472 (67.4)	261 (48.9)	< 0.01
	Often or always	228 (32.6)	273 (51.1)	
Time working as a pilot	Up to 10 years	324 (46.3)	181 (34.1)	< 0.01
	11 to 20 years	192 (27.4)	206 (38.8)	
	21 to 30 years	114 (16.3)	115 (21.8)	
	31 years or more	70 (10)	31 (5.3)	
Time working in the night shift	Less than 1 year	404 (57.7)	272 (50.9)	< 0.01
	1 to 5 years	130 (18.6)	90 (16.9)	
	6 to 10 years	81 (11.6)	83 (15.5)	
	11 to 15 years	27 (3.8)	41 (7.7)	
	16 years or more	58 (8.3)	48 (9.0)	
Quality of place for resting in the plane	≥ 11 points	353 (51.1)	228 (43.3)	< 0.01
	≤ 10 points	337 (48.8)	299 (56.7)	
End of work in the night shift	Before 5:00 am	358 (55.0)	239 (47.2)	0.02*
	Between 5:01 am and 8:00 am	229 (35.2)	194 (38.3)	
	Between 8:01 am and 12:00 pm	60 (9.2)	67 (13.3)	
	Between 12:01 pm and 4:00 pm	4 (0.6)	6 (1.2)	
Need for recovery after work	Less need	315 (45.0)	77 (14.4)	< 0.01
	Moderate need	228 (32.6)	200 (37.5)	
	Greater need	157 (22.4)	257 (48.1)	
Occupational stress	Low job strain	79 (11.3)	113 (21.2)	< 0.01
	Passive work	180 (25.7)	99 (18.5)	
	High job strain	287 (41.0)	147 (27.5)	
	Active work	154 (22.0)	175 (32.8)	

* Fisher's exact test

In the bivariate model, the following variables were associated with low or moderate work ability: insufficient sleep, poor sleep, high chance for development of obstructive sleep apnea syndrome, unintentional naps during work, symptoms of insomnia, excessive sleepiness, high perception of fatigue, international flight routes, 66 or more monthly flight hours, up to nine days off per month, seven or more consecutive days of work, frequent operational delays, career of 11 to 20 years working as a pilot, 11 to 15 years working on the night shift, assessment of the place for rest on the plane as poor, end of night shift between 8:01 am and 12:00 pm, and moderate and greater need for recovery after work. Factors of protection for low or moderate work ability included: being international captain and domestic copilot, working as pilot for 21 to 30 years, and having passive work with high job strain (Table 2).

**Table 2:** Gross and adjusted prevalence ratios of factors associated with moderate or low work ability of regular aviation pilots. Brazil, 2017.

Variable		Bivariate	Adjusted multiple*
		PR (95%CI)	PR (95%CI)
Weekly physical activity time			
	More than 150 minutes	1	
	Up to 150 minutes	1.22 (1.03–1.44)	
Perception of chronotype			
	Indifferent	1	
	Morningness or extreme morningness	1.26 (0.92–1.72)	
	Eveningness or extreme eveningness	1.29 (0.95–1.75)	
Perception of sufficient sleep time			
	Yes	1	1
	No	1.61 (1.36–1.92)	1.29 (1.06–1.57)
How well do you think you sleep			
	Very well or well	1	
	Neither well nor poorly	1.57 (1.31–1.90)	
	Fairly or very poorly	2.20 (1.73–2.82)	
Obstructive sleep apnea syndrome			
	Low chance	1	
	High chance	1.55 (1.29–1.88)	
Sleepiness (Epworth)			
	Low sleepiness	1	
	Excessive sleepiness	1.41 (1.19–1.68)	
Perception of fatigue			
	Lower fatigue	1	1
	Higher fatigue	1.87 (1.56–2.23)	1.51 (1.24–1.84)
Unintentional nap during work			
	No	1	
	Yes	1.75 (1.46–2.11)	
Symptoms of insomnia			
	Without symptoms	1	
	With symptoms	1.79 (1.49–2.15)	
Current function			
	International captain	1	
	Domestic captain	0.73 (0.54–0.99)	
	International copilot	0.85 (0.49–1.44)	
	Domestic copilot	0.58 (0.43–0.81)	
Flight routes			
	Domestic	1	
	International	1.42 (1.11–1.84)	
Monthly flight hours			
	Up to 65 hours	1	1
	66 hours or more	1.27 (1.07–1.51)	1.22 (1.01–1.46)
Average days off per month			
	10 days or more	1	1
	Up to 9 days	1.45 (1.21–1.74)	1.27 (1.04–1.55)
Maximum number of consecutive days of work			
	Up to 6 days	1	
	7 days or more	1.30 (1.06–1.59)	
Maximum number of consecutive nights of work			
	One or two nights	1	
	Three or four nights	1.05 (0.83–1.35)	
	Five nights or more	1.18 (0.91–1.55)	
Frequency of operational flight delays			
	Never, rarely or sometimes	1	1
	Often or always	1.53 (1.29–1.81)	1.23 (1.02–1.48)
Time working as a pilot			
	Up to 10 years	1	
	11 to 20 years	1.44 (1.18–1.76)	
	21 to 30 years	1.40 (1.10–1.77)	
	31 years or more	0.85 (0.58–1.25)	
Years working night shifts			
	Less than 1 year	1	
	1 to 5 years	1.02 (0.81–1.29)	
	6 to 10 years	1.26 (0.98–1.60)	
	11 to 15 years	1.49 (1.08–2.08)	
	16 years or more	1.13 (0.83–1.53)	
Quality of place for resting in the plane			
	≥ 11 points	1	
	≤ 10 points	1.19 (1.00–1.43)	
End of work in the night shift			
	Before 5:00 am	1	
	Between 5:01 am and 8:00 am	1.15 (0.95–1.38)	
	Between 8:01 am and 12:00 pm	1.32 (1.00–1.73)	
	Between 12:01 pm and 4:00 pm	1.49 (0.66–3.37)	
Need for recovery after work			
	Less need	1	
	Moderate need	2.38 (1.83–3.09)	
	Greater need	3.16 (2.45–4.07)	
Occupational stress			
	Low job strain	1	
	Passive work	0.61 (0.46–0.79)	
	High job strain	0.57 (0.45–0.74)	
	Active work	0.91 (0.72–1.14)	

* Model adjusted for sex and age. Hoc curve 0.71, 95%CI 0.68–0.74.

While in the multiple model adjusted for sex and age, the variables that presented risk of prevalence for low or moderate work ability were: insufficient sleep, higher perception of fatigue, 66 or more monthly flight hours, less than 10 days off per month, and frequent operational delays (Table 2).

## DISCUSSION

We observed a relevant percentage of pilots with moderate or low work ability, which is an important aspect, because the study population consisted of young adults and the main factors associated with decreased work ability are related to work organization. Sluiter^24^ points out that functions with high demand of work, as in the case of airline pilots, impair work ability, even in younger workers^4^ ^,^ ^24^. In this study, we observed higher proportion of pilots with an active work, that is, with high demand and high control, with moderate or low work ability, in relation to great or good work ability.

When comparing the prevalence of moderate or low work ability of this study with others that used the same evaluation tool, we found that it was very high. Marqueze and Moreno^12^, studying higher education professors with similar mean age to that of the pilots, found a prevalence of 13%. It is worth mentioning that the professors worked on the day, evening, and night shifts until 11 pm. Two studies with nursing professionals observed prevalence of moderate or low work ability lower than or equal to that of the pilots in this article. The nursing professionals’ mean age was 39.4 years and 41.3 years and they also worked in shifts and at night. The prevalence of moderate or low work ability was 35%^5^ and 43.3%^21^. It should be pointed out that the study of Prochnow et al.^21^ used the cut-off point established by Tuomi et al.^25^, which is proposed for workers aged from 45 years. While the study of Silva et al.^5^ used the cut-off point of Tuomi et al.^25^ and that of Kujala et al.^10^, according to the age of respondents. However, in this study, we employed only the cut-off point proposed by Kujala et al.^10^, because it is indicated for younger workers, as in the case of the sample under study (74.2% of the pilots were aged up to 45 years). In the study of Kujala et al.^10^, with Finnish workers of different occupational areas, the prevalence of moderate and low work ability was also lower than in our study (39.0% in men)^10^.

The respondents’ age is an aspect that needs to be highlighted, because, on average, the pilots studied were young adults, but already showed signs of impaired work ability. According to Tuomi et al.^25^, functional aging is expected as chronological age advances; however, in this study age was not associated with low or moderate work ability. In the study of Marqueze and Moreno^12^, the authors also did not observe this relationship and said that functional aging is not necessarily related to chronological aging, but mainly to working conditions, which seems to be the same situation for the pilots in the study. As described previously, airline pilot work is complex and requires different skills and activities from the professional, mainly mental requirements. These work requirements can trigger chronic and acute physiological responses, psychological reactions, and behavioral changes, with the possibility of decreased work ability^8^.

It was found that insufficient sleep was a factor associated with moderate or low work ability. According to Melo and Neto^14^, sleep deprivation in pilots is a reflection of irregular workdays. It is known that sleep restriction for a prolonged time can cause fatigue, leading to decreased level of alert and increased irritability, among other negative effects^13^ ^,^ ^15^ ^,^ ^20^ ^,^ ^22^. Fatigue was also one of the factors associated with moderate or low work ability. This result corroborates the study of Silva et al.^5^, in which the authors found that the higher the fatigue, the lower the work ability.

Sleep deprivation increases the possibility of unintentional naps during the flight^13^, and one of the factors that lead to this greater sleep deprivation is the reversal of the sleep-wake cycle because of exposure to work in irregular shifts^7^ ^,^ ^11^ ^,^ ^20^ ^,^ ^23^. As verified, perception of insufficient sleep increased by almost 30% the prevalence of moderate or low work ability. Most pilots reported starting the morning shift before 5:00 am and ending the evening shift after 10:00 pm, in addition to often working the night shift, thus affecting negatively the time available for sleep. According to Goode^6^, pilots should have the opportunity to sleep at least eight hours in the rest time. This could improve work ability, as well as avoid incidents.

In this study, increased flight hours (≥66 hours/month) was a factor associated with moderate or low work ability. Roach et al.^22^ and Lamond et al.^11^ claim that short workweeks (less than 40 hours/week), although less extensive, have shorter rest time between workdays, which leads to fatigue. While average workweeks (40–61 hours/week) and long workweeks (over 62 hours/week) have longer rest time between work periods. However, more extensive work periods are harmful to health, as verified in this study. Thus, the authors recommend increased rest time between work periods, as well as shorter work periods, given the complexity and volume of the pilots’ work^11^ ^,^ ^22^. It is pointed out that the flight hours reported by study participants did not exceed those provided for in law (80 hours/month – Law 13.475 of August 28, 2017^c^). However, a study limitation is that we collected no information on total workday (timetables for report, for engine shutdown, and duration of operational delays); therefore, actual flight hours are less than total work time.

Having less than 10 days off per month was also a factor associated with low or moderate work ability. Working for a long consecutive time, without time off, and with an extensive work period, considering the high cognitive demands of the profession, can lead to a situation of fatigue and, consequently, decreased work ability^5^ ^,^ ^7^ ^,^ ^9^.

Frequent operational delays have also been associated with low or moderate work ability. According to Law 13,475^c^, a workday is counted from the time of report to the workplace, which should be at least 30 minutes before the flight, and ends 30 minutes after the final stop of engines for domestic flights and 45 minutes for international flights. Therefore, it is understood that a delay or even small delays could increase the workday of the pilot, who is already working on the limit^6^ ^,^ ^7^, which leads to increased fatigue.

In the bivariate model, in addition to the factors already reported in the multiple model, other aspects related to sleep were also associated with low or moderate work ability. These factors include: poor perception of sleep, excessive sleepiness, unintentional naps during work, and symptoms of insomnia. This result reinforces how sleep problems may influence work ability^5^. According to Itani^9^, harmful effects were observed in the health of pilots working in irregular shifts and with poor conditions of rest, such as inadequate place for rest on the plane and little time to sleep.

Aspects concerning work organization were also associated with low or moderate work ability in the bivariate model. Among them: international flight routes, consecutive work for five nights or more, longer career and more time on the night shift, a place with poor quality for rest on the plane, end of night shift between 12 pm and 4 pm, and need for longer recovery. These factors indicate that organizational aspects that directly affect the rest of pilots can lead to early functional aging, which requires actions to minimize this impact, as pointed out previously.

While aspects related to occupational stress were factor of protection; however, prolonged exposure of workers to work stressors may be related to the emergence of musculoskeletal symptoms and early functional aging^25^. Menegon^d^ also found that working time and workplace had important role in decreasing the work ability of individuals working in structural assembly of aircraft. The data presented above corroborate the hypothesis that work organization has major influence on work ability, and that working hours may be central in this discussion.

This study presents some limitations, which do not allow generalizations of results found. We used convenience sampling, since only pilots associated with the ABRAPAC were invited to participate in the study. However, internal validity was high (99% sample power), and around 25% of the total Brazilian pilots registered in the Anac^a^ composed the sample. Another limiting factor refers to the insomnia symptoms questionnaire. This questionnaire had no validation and translation to Portuguese, which may be considered a bias in the study. However, it should be noted that this questionnaire has been used in some Brazilian studies^16^, and in this study showed a high degree of reliability and consequent validation verified by Cronbach's alpha.

Additionally, the study design (cross-sectional) does not allow to determine the temporal relationship of the variables studied. However, such studies are fundamental to know the sociodemographic, work, and sleep characteristics of this population and may be a reference for future studies. With the significant increase in demand for air travel, the decreasing number of pilots, and the consequent overworking, the risk of deleterious effects to the health of this professional category is considerable and would generate great impact on the economy. Therefore, disease prevention and health promotion actions are essential to avoid this situation. It is pointed out that there are no records of previous Brazilian studies similar to this, as to sample size and diversity of pilots, since they worked in the five major commercial airlines in the country.

Finally, we can conclude that work organization was a determining factor for decreased work ability, especially concerning aspects related to rest and its repercussions on the sleep of pilots.

## Footnotes

a Agência Nacional de Aviação Civil, Superintendência de Acompanhamento de Serviços Aéreos, Gerência de Acompanhamento de Mercado. Anuário do Transporte Aéreo 2014. Brasília (DF):ANAC; 2014.

b Marqueze EC, Diniz DHMD, Nicola AC. Fadiga crônica, condições de trabalho e saúde em pilotos brasileiros. São Paulo: Associação Brasileira de Pilotos da Aviação Civil – ABRAPAC; 2014. Available from: https://pilotos.org.br/press-release-pesquisa-de-fadiga/

c Brasil. Lei n° 13.475, de 28 de agosto de 2017. Dispõe sobre o exercício da profissão de tripulante de aeronave, denominado aeronauta; e revoga a Lei n° 7.183, de 5 de abril de 1984. Brasília (DF); 2017 [cited 2017 Dec 13]. Available from: http://www.planalto.gov.br/ccivil_03/_ato2015-2018/2017/lei/L13475.htm

d Menegon FA. Atividade de montagem estrutural de aeronaves e fatores associados à capacidade para o trabalho e fadiga [these]. São Paulo: Faculdade de Saúde Pública da Universidade de São Paulo; 2011.
